# The association between cognitive tests and stages of cognitive decline in Caribbean Hispanics

**DOI:** 10.1002/alz.092997

**Published:** 2025-01-03

**Authors:** Katalina F McInerney, Andrew F Zaman, Michael B. Prough, Pedro R. Mena, Larry D. Adams, Jose Javier Sanchez, Vanessa C Rodriguez, Glenies S Valladares, Katrina Celis, Kara L. Hamilton‐Nelson, Farid Rajabli, Gary Beecham, Briseida E. Feliciano‐Astacio, Jeffery M. Vance, Margaret A Pericak‐Vance, Michael L. Cuccaro

**Affiliations:** ^1^ University of Miami Miller School of Medicine, Miami, FL USA; ^2^ John P. Hussman Institute for Human Genomics, University of Miami Miller School of Medicine, Miami, FL USA; ^3^ Dr. John T. Macdonald Foundation Department of Human Genetics, University of Miami Miller School of Medicine, Miami, FL USA; ^4^ Puerto Rico Alzheimer’s disease and Related Disorders Initiative, Caguas, Puerto Rico USA; ^5^ Universidad Central del Caribe, Bayamón, PR USA; ^6^ Department of Neurology, University of Miami Miller School of Medicine, Miami, FL USA; ^7^ 1501 NW 10th Avenue, Miami, FL USA

## Abstract

**Background:**

The utility of neuropsychological measures commonly used to diagnose individuals with suspected MCI or AD was recently explored in European cohorts, however their utility in Caribbean Hispanic (CH) populations is not well understood.

**Method:**

Our sample consisted of 507 CH individuals from ongoing studies of Puerto Rican and Cuban American older adults (74%% Female, mean age = 74.1, mean education = 12.7 years, 209 Unaffected, 185 MCI, 113 with AD) who completed cognitive measures from the NACC UDS2 battery (Logical Memory, Semantic Fluency [animals and vegetables], and Digit Span). Participants were clinically adjudicated as unaffected, MCI, or AD, and had a CDR global score ≤1. Individuals with disorders known to affect cognition were excluded. An ordinal regression explored which cognitive tests were most strongly associated with clinical diagnoses. We ran this analysis again on a subset of participants who also completed the Trails Making Test (n = 242, 103 unaffected, 97 MCI, 42 AD). We examined both the log‐odds estimate and Wald value.

**Result:**

An ordinal regression on the full sample showed that the tests associated with differences in clinical diagnosis in order of their predictive value are Delayed Logical Memory (log‐odds = 1.29, Wald = 40.1, p<0.001), Digit Span Backwards (log‐odds = 0.70, Wald = 25.2, p<0.001), Animals (log‐odds = 0.44, Wald = 12.0, p<0.001), Vegetables (log‐odds = 0.32, Wald = 7.3, p<0.01), and Digit Span Forwards (log‐odds = 0.24, Wald = 6.3, p = 0.012). On the subset that completed the Trails Making Test, Trails B emerged as most predictive (log‐odds = 0.70, Wald = 26.8, p <0.001), but Vegetables (p = 0.26), Logical Memory Immediate (p = 0.43), and Trails A (p = 0.70) were not significant predictors of differences in clinical diagnosis in this smaller group.

**Conclusion:**

In our sample of CH individuals, we found performance on measures of delayed contextual memory (Logical Memory), working memory (Digit Span Backwards and Trails B) and semantic fluency (Animals) to be most useful in differentiating individuals clinically diagnosed as unaffected, MCI, or AD. These results highlight possible performance patterns specific to CH that can inform adjudication efforts with this population.

